# Calcium sensing receptor regulate claudin-14 via PKA-STAT3 pathway in rat model of nephrolithiasis

**DOI:** 10.3389/fphar.2024.1477122

**Published:** 2024-12-04

**Authors:** Peiyue Luo, Tao Chen, Liying Zheng, Junrong Zou, Jun Zou, Wei Li, Qi Chen, Le Cheng, Biao Qian

**Affiliations:** ^1^ The First Clinical College, Gannan Medical University, Ganzhou, Jiangxi, China; ^2^ Department of Urology, The First Affiliated hospital of Gannan Medical University, Ganzhou, Jiangxi, China; ^3^ Key Laboratory of Urology and Andrology of Ganzhou, Ganzhou, Jiangxi, China; ^4^ Department of Graduate, The First Affiliated Hospital of Gannan Medical University, Ganzhou, Jiangxi, China

**Keywords:** kidney stones, calcium-sensitive receptor, claudin-14, calcium oxalate, signal transducer and activator of transcription 3

## Abstract

**Background:**

The calcium-sensitive receptor (CaSR) has been identified as a key factor in the formation of kidney stones. A substantial body of research has illuminated the function of CaSR in stone formation with respect to oxidative stress, epithelial injury, crystal adhesion, and stone-associated proteins. Nevertheless, as a pivotal molecule in renal calcium excretion, its pathway that contributes to stone formation by regulating calcium supersaturation remains underexplored.

**Methods:**

An in vitro rat calcium oxalate kidney stone model was established through the co-cultivation of calcium oxalate monohydrate (COM) with NRK-52E cells, while an in vivo model was constructed using the ethylene glycol method. Subsequently, the level of the CaSR-claudin-14 pathway was determined. To further elucidate the molecular pathway of CaSR-mediated regulation of claudin-14, drugs were selectively added to the in vitro and ex vivo kidney stone models, and the expression of claudin-14 and the levels of stone formation were detected. Moreover, the direct regulation of claudin-14 by CaSR with STAT3 serving as a transcription factor was examined via the dual luciferase assay. Eventually, a Cldn-14 knockout rat model and a model of kidney stone induction by ethylene glycol were generated using CRISPR-Cas9 technology to further clarify the role of claudin-14 in the CaSR-regulated formation of kidney stones.

**Results:**

In vitro and in vivo observations revealed that calcium oxalate induces high expression of CaSR-claudin-14. Specifically, CaSR regulates claudin-14 expression through phosphorylation modification of STAT3 via protein kinase A (PKA). In vitro, the intervention of PKA and STAT3 reversed the elevated claudin-14 levels and stone formation induced by CaSR. Finally, we generated cldn-14 knockout rats using CRISPR-Cas9 technology and observed that ethylene glycol still induced stone formation in these animals. Nevertheless, the specific activation or inhibition of CaSR demonstrated no notable impact on stone formation.

**Conclusion:**

The results of our study indicate that calcium oxalate crystals induce the activation of the pro-stone pathway of CaSR. That is, activated CaSR regulates claudin-14 levels via the PKA-STAT3 pathway, which further promotes calcium salt stone formation. The role of CaSR in the regulation of stone homeostasis is further enriched.

## Introduction

Nephrolithiasis is a prevalent affliction of the urinary tract globally (Hill et al., 2022; Zeng et al., 2017). Moreover, the postoperative recurrence incidence of nephroliths is notably elevated, typically reaching 20%–45% within 2 years and 50%–70% within 5 years (Kavoussi et al., 2023). Although the majority of patients who undergo clinical interventions can currently achieve satisfactory therapeutic results, the recurrence tendency of nephrolithiasis remains unknown. Recurrent stone formation not only exacerbates patient discomfort and imposes economic burden but also predisposes individuals to complications such as infection and renal impairment, necessitating nephrectomy in severe cases (Rule et al., 2009; Rivera and Krambeck, 2016). Consequently, the effective prevention and management of nephrolithiasis have emerged as imperatives warranting investigation, with the elucidation of its pathogenesis serving as the cornerstone for resolution.

Calcium-sensitive receptor (CaSR), a G-protein-coupled receptor, was initially identified in bovine thyroid glands (Brown et al., 1993). It is predominantly expressed in the thyroid gland and renal tubules, and exerts biological effects through the activation of intracellular signaling pathways by G proteins (Hebert et al., 1997). CaSR is extensively distributed throughout all segments of renal tubules and exerts a range of functions. In the proximal tubule, CaSR suppresses calcium reabsorption, promotes phosphate reabsorption, and inhibits citrate reabsorption (Ba et al., 2003; Walker et al., 2018). In the collecting ducts, CaSR inhibits the expression of the water channel protein aquaporin 2 (AQP2), leading to urine dilution, and increases the activity of the proton pump, resulting in urine acidification (Bustamante et al., 2008; Renkema et al., 2009; Casare et al., 2014). In the distal tubule, CaSR inhibits the calcium pump, plasma membrane Ca -ATPase (PMCA), which in turn inhibits calcium reabsorption (Blankenship et al., 2001). Similarly, in the thick ascending limb (TAL), CaSR inhibits calcium reabsorption and promotes urinary calcium excretion through mechanisms such as inhibition of sodium-potassium-chloride cotransporter protein (NKCC2) and low conductance potassium channels (ROMK) and promotion of claudin-14 expression (Gamba and Friedman, 2009; Dimke et al., 2013). In conclusion, CaSR plays a dual role in renal stone formation. On one hand, it inhibits water reabsorption, promotes proton secretion, and inhibits citrate reabsorption, thereby exerting an inhibitory effect on the process of renal stone formation. On the other hand, it has been demonstrated to facilitate urinary calcium excretion, thereby contributing to the formation of renal stones.

The primary pathway for calcium reabsorption in the renal tubule is the paracellular pathway, which is mediated by a complex formed by the paracellular pore composed of tight junction proteins, specifically claudin proteins. Among these, claudin-16 and claudin-19 constitute cation channels that mediate the reabsorption of divalent cations, which are required for calcium and magnesium ion reabsorption (Simon et al., 1999; Konrad et al., 2006). Claudin-14 is a crucial component of the paracellular pathway in TAL cells and can modulate calcium reabsorption by regulating the permeability of the paracellular cation channels constituted by claudin-16 and claudin-19 (Gong et al., 2015) A number of studies have demonstrated the role of claudin-14 in stone formation (Dimke et al., 2013; Thorleifsson et al., 2009). It has been demonstrated that claudin-14 is regulated by CaSR, which alters renal excretion of calcium ions in response to changes in serum calcium levels (Dimke et al., 2013). Our previous study demonstrated that CaSR and claudin-14 expression was enhanced during the modeling of kidney stones in rats (Qian et al., 2022). Moreover, the function of the CaSR-claudin-14 pathway in maintaining calcium balance and the formation of renal stones *in vivo* has been substantiated (Gong et al., 2012).

But the mechanism by which CaSR activates claudin-14 remains unclear. It has been pointed out that CaSR can regulate the expression of claudin-14 through miRNAs, and although it has been reported that the regulation of claudin-14 by CaSR is achieved through miRNA-9 (Gong et al., 2012), we wonder whether CaSR can affect the expression of *cldn14* by regulating its transcription factors. Therefore, we predicted the promoter sequence of the *cldn14* gene (http://bioinfo.life.hust.edu.cn/hTFtarget#!/prediction) and found that signal transducer and activator of transcription 3 (STAT3) had the highest score among all known transcription factors. In addition, CaSR acts as a G-protein-coupled receptor. Activated CaSR activates downstream signaling pathways mediated by G-protein coupling. In the G protein-coupled receptor-mediated signaling pathway, protein kinase A (PKA) is an important downstream effector protein. It has also been shown in the literature that CaSR activates PKA, and activation of STAT3 by PKA has been demonstrated in a variety of cells (Li et al., 2019; Macías-García et al., 2019). Therefore, we speculate that the regulation of claudin-14 by CaSR may be achieved through the CaSR-PKA-STAT3-claudin-14 pathway, and we further speculate that CaSR-PKA-STAT3-claudin-14 may be activated during the stone formation process, thereby inhibiting calcium reabsorption and promoting urinary calcium excretion, leading to further formation of kidney stones. STAT3 may play a very important role in the activation of claudin-14.

Therefore, this study aimed to elucidate the relationship between the CaSR-claudin-14 pathway and stone formation, and to clarify how CaSR modulates claudin-14 expression, exploring potential intervention points for kidney stones.

## Materials and methods

### Materials

R568 (GC17700), NPS-2143 (GC16943), 8-Bromo-cAMP (GC16929), H 89 2HCl (GC10074), IL-6 (GP20521), and STAT3-IN-1 (GC37688) were purchased from GlpBio Technology Inc. (United States). Anti-beta actin monoclonal antibody (66009-1-Ig), anti‐mouse immunoglobulin G (IgG) and anti‐rabbit IgG horseradish peroxidase‐conjugated secondary antibodies were purchased from Proteintech Group, Inc. (United States). An anti-CaSR monoclonal antibody (73303S), an anti-phospho-STAT3 monoclonal antibody (9145S), and an anti-phospho-PKA substrate monoclonal antibody (9624S) were obtained from Cell Signaling Technology (Danvers, MA, United States). An anti-claudin-14 polyclonal antibody (36–4,200) was purchased from Thermo Fisher Scientific (Waltham, MA, United States).

### Cell culture and treatment

NRK-52E cells were obtained from Procell Life Science Technology (Wuhan, China) and cultured in Dulbecco’s modified eagle medium (DMEM) (Thermo Fisher Scientific, United States) supplemented with 10% fetal bovine serum (Thermo Fisher Scientific, United States), 100 U/mL penicillin (Beijing Solarbio Science&Technology Co.,Ltd., China), and 100 mg/mL streptomycin (Beijing Solarbio Science&Technology Co.,Ltd., China). The medium was refreshed every 2–3 days, and passaging was conducted when the cell confluence reached approximately 80%. Cultures were incubated in a humidified environment at 37°C with 5% CO2/95% O2. Calcium oxalate monohydrate (COM) were synthesized following established protocols from the literature (Chen et al., 2010). Upon entering the logarithmic growth phase, the cells were exposed to COM crystals and treated with the relevant drugs for 6 h according to the cell groups. Subsequently, the cells were harvested for further analysis.

### Experimental animals and protocol

The study was approved by the Institutional Animal Care and Use Committee of Gannan Medical University (Ganzhou, China). Six-week-old male Wistar rats (200–225 g) were obtained from the Experimental Animal Center of Gannan Medical University (Ganzhou, China, NO.202230760). Cldn-14 gene knockout heterozygous rats were purchased from Cyagen Biosciences, Inc. (Jiangsu, China, NO.44826100001973). Animals were housed under standard conditions with a constant temperature of 24°C ± 2°C and humidity of 55% ± 5%, having free access to both food and distilled water. The experiment was conducted in two phases. In the first phase, wild-type and cldn-14 gene knockout rats were acclimatized for 1 week before being randomly allocated into four groups: The negative control (NC) group (drank ddH₂O freely and received intramuscular saline as a control), the ethylene glycol (E.G.,) group (drank 1% E.G., and received intramuscular saline), the calcium-sensitive receptor agonist (CaSRA) group (drank 1% E.G., and was intramuscularly injected with R568 at 0.12 mg/kg), and the calcium-sensitive receptor inhibition (CaSRI) group (drank 1% E.G., and was intramuscularly injected with NPS2134 at 0.15 mg/kg). Two additional wild-type groups were added in the second phase, based on the, E.G., group and CaSRA group: the protein kinase A inhibition (PKAI) group, which received intramuscular injections of the PKA inhibitor H892HCL (0.5 mg/kg) in conjunction with the CaSRA group treatment, and the signal transducer and activator of transcription 3 inhibition (STAT3I) group, which received intramuscular injections of the STAT3 inhibitor Stattic (3.75 mg/kg) alongside the CaSRA group treatment. All drug intramuscular injection concentrations were selected according to the drug instructions and pre-experimental results. The animals were sacrificed by rapid cervical dislocation followed by injections of 4°C physiological saline into the bilateral renal veins, aiming to reduce blood residue within the kidney tissues. Kidney tissues were extracted and individually preserved in liquid nitrogen and a fixation solution for subsequent analysis via Western blot and pathological examination.

### Pathological examination

Paraffin-embedded kidney sections (4 μm) were baked at 65°C for 2 h and then deparaffinized by immersion in xylene and gradient alcohol (100, 95, 90% and 80%) for HE and calcium staining, respectively (Pizzolato). Examinations were carried out utilizing an optical microscope and a polarized light microscope, with assessments of renal tubular crystal deposition and renal injury scoring. Kidney tissue samples intended for electron microscopy were sectioned into 1–3 mm³ blocks, fixed in 2.5% glutaraldehyde, and osmotically embedded. Subsequently, ultrathin sections (60–80 nm) were stained and examined using transmission electron microscopy.

### Immunohistochemical staining

Paraffin-embedded kidney sections were dewaxed according to the conventional pathological sectioning method, and then the sections were placed in citric acid buffer (pH = 6.0) (Beijing Solarbio Science&Technology Co.,Ltd., China) at 90°C–98°C and boiled for 10 min. Following natural cooling, the sections were incubated in 3% H2O2 solution (Beijing Solarbio Science&Technology Co.,Ltd., China) at 37°C for 15 min to remove endogenous peroxidase. After blocking nonspecific protein binding with 5% bovine serum albumin (Thermo Fisher Scientific, United States) at 37°C for 30 min, the sections were incubated overnight with primary antibodies against CaSR (1:500) and claudin-14 (1:300) at 4°C in a humid environment. Subsequently, the sections were incubated with a secondary antibody that matched the source of the primary antibody at 37°C for 30 min and developed using diaminobenzidine. Finally, the tissue sections were dehydrated and cleared with gradient alcohol (80, 90, 95% and 100%) and xylene, followed by sealing with neutral resin before observation under a microscope.

### Western blotting analysis

Cell lysates were prepared by adding 10 μL of protease inhibitor (GlpBio Technology Inc.,United States) and 10 μL of phosphatase inhibitor (GlpBio Technology Inc.,United States) to 1 mL of RIPA buffer (Shanghai Beyotime Biotech Inc., China). After washing with PBS (GlpBio Technology Inc.,United States), the cells were lysed with lysis buffer for 30 min (the tissue was prepared as a cell homogenate at 4°C and then lysed). Then, the supernatant was collected by centrifugation at 12,000 × g for 10 min at 4°C. The protein sample concentration was quantified using the BCA method and adjusted to 2 mg/mL. Subsequently, the protein sample was combined with SDS‒PAGE sample loading buffer and incubated at 95°C for 5 min. Subsequently, protein samples were electrophoresed using a sodium dodecyl sulfate‒polyacrylamide gel containing 10% acrylamide for separation. The separated proteins were subsequently transferred to a PVDF membrane by constant-current electrotransfer. After the membranes were blocked with 5% skim milk powder at room temperature for 2 h, they were incubated with antibodies against CaSR (1:2,500), p-PKA (1:1,000), p-STAT3 (1:1,000), and claudin-14 (1:1,000) at 4°C overnight. After being washed with TBST, the membrane was incubated with the corresponding secondary antibody (1:2,500) labeled with horseradish peroxidase at room temperature for 2 h and then washed with TBST again. Finally, the proteins were detected by enhanced chemiluminescence.

### Intracellular cAMP measurement

After treatment, the cells were washed with PBS, and the cells were fully lysed by adding appropriate amount of cell lysis solution. The cell supernatant was collected by centrifugation and the cAMP level was determined according to the instructions of cAMP ELISA kit (Wuhan Elabscience Biotechnology Co.,Ltd. China).

### Dual-luciferase assay

Assays were performed according to the manufacturer’s instructions (Promega Corp.). NRK-52E cells were seeded into 24-well plates (2 × 104 cells per well). Upon reaching 70% confluence, the cells were co-transfected with pGL4 constructs harboring firefly luciferase and renin luciferase downstream of the cldn-14 promoter. After 4–6 h, the DMEM supplemented with 10% FBS was replaced, followed by a 24-h incubation at 37°C in a 5% CO2 incubator. Cell lysates were generated by adding 100 μL of PLB to each well. Next, 100 μL of LAR II was predisposed into a black opaque 96-well plate. Then, 20 μL of the cell lysate was added, mixed, and transferred to a luminescence detector, after which the firefly luciferase activity was measured. Subsequently, 100 μL of Stop and Glo^®^ reagent was dispensed, gently agitated, and returned to the luminescence detector. At this juncture, renin luciferase activity was assessed, and the firefly luciferase activity/renin luciferase activity ratio was computed for each well.

### Statistical analysis

The data are presented as the mean ± standard error. All the data reported stem from measurements taken from a minimum of three independent samples. The normality of the distribution of the data was evaluated using the Shapiro‒Wilk test. Student’s t-test was utilized for comparisons between pairs of groups, whereas one-way analysis of variance (ANOVA) was applied for comparisons encompassing three or more groups to discern significant disparities. A *p*-value less than 0.05 was considered to indicate statistical significance.

## Results

### Calcium oxalate increases expression of CaSR and claudin-14 *in vivo* and *in vitro*

**FIGURE 1:**
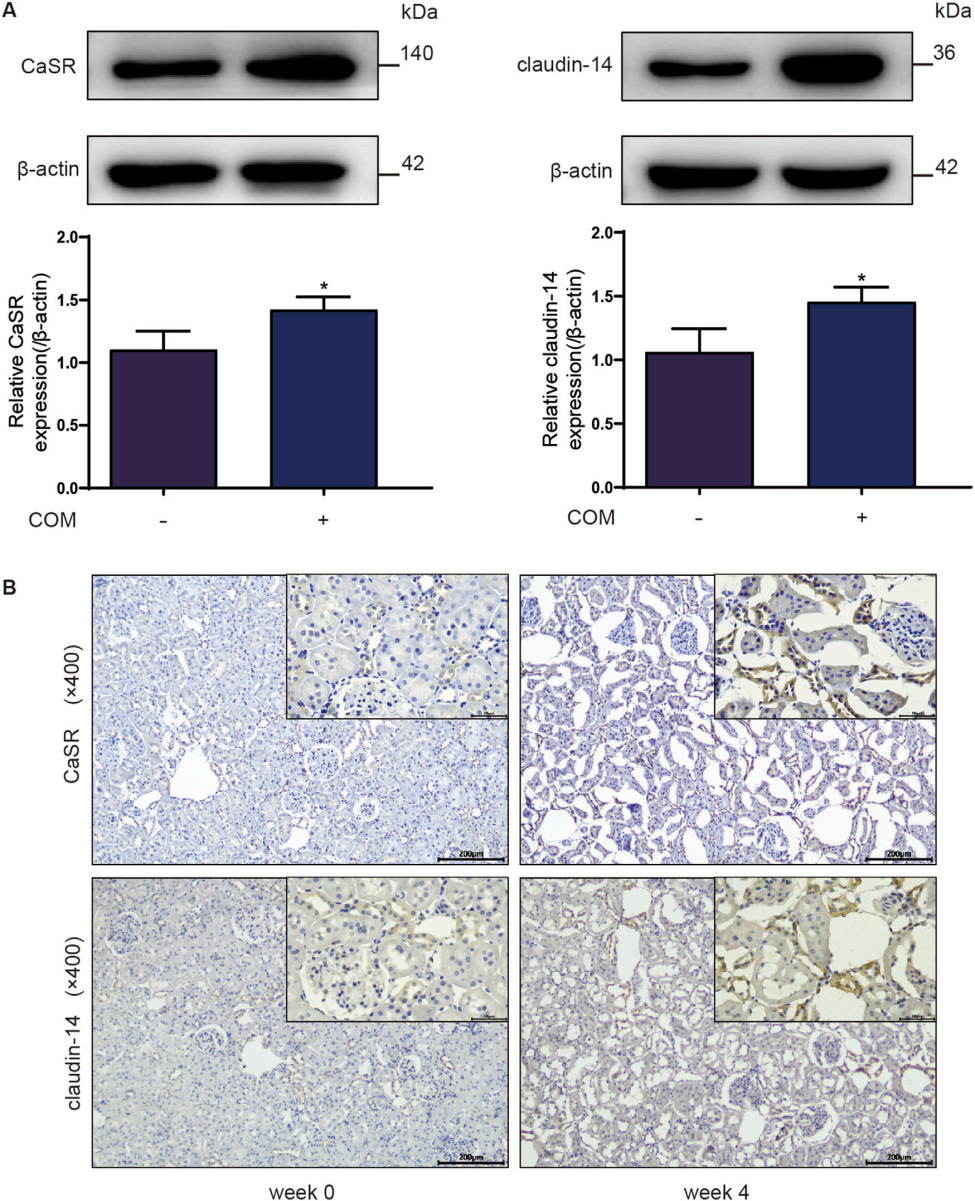
Protein expression of CaSR and claudin-14 in a calcium oxalate environment. **(A)** Protein expression of CaSR and claudin-14 in NRK-52E cells. The data are presented as the mean ± standard deviation of three independent experiments. *p < 0.05 vs the control group. Statistical analyses were performed by two-tailed unpaired t-tests. **(B)** Immunohistochemical distribution of CaSR and claudin-14 protein expression in the rat kidney after 4 weeks of ethylene glycol treatment. The left column comprises representative images showing the expression of CaSR and claudin-14 in 0-week-old rats, and the right column comprises representative images showing the expression of CaSR and claudin-14 in 4-week-old rats. COM, calcium oxalate monohydrate; CaSR, calcium-sensing receptor.

To explore whether the CaSR-claudin-14 pathway is activated during stone formation, calcium oxalate kidney stone models were established and evaluated both *in vivo* and *in vitro*. Figure 1A illustrates the changes in CaSR and claudin-14 expression in NRK-52E cells following exposure to 134 µg COM crystals/cm^2^ of cells for 6 h. The results identified a specific band at 140 kDa, which exhibited a statistically significant elevation following COM induction. It is established that CaSR functions as an obligate homodimer (Gao et al., 2021). Western blot analysis conducted under non-reducing conditions revealed the presence of double bands at 140 and 200–235 kDa, indicative of the monomeric and dimeric forms of CaSR, respectively. However, the present study was conducted under reducing conditions, which resulted in the observation of a single band with a molecular weight of 140 kDa. Moreover, the results of the assay demonstrated that COM induced an increase in claudin-14 expression. Notably, there was a strong correlation between the levels of CaSR and claudin-14 under COM induction.

Investigators further assessed the expression levels of these two proteins in kidney stones *in vivo*. Rats were freely provided with 1% E.G., and renal tissue was extracted weekly for calcium oxalate crystal detection and analysis of CaSR and claudin-14 expression levels. As shown in the immunohistochemistry results in Figure 1B, CaSR and claudin-14 were expressed at low levels in renal tissue under normal conditions (week 0). Starting from week 3, we began to detect positive staining within the cytoplasm of some distal renal tubular epithelial cells, and this staining continued to intensify over time. Simultaneously, the emergence of calcium oxalate crystals within the renal tubules (mainly in distal tubules) of the rats was observed during this time period. Furthermore, the accumulation of these crystals increased progressively over time. In summary, these findings indicate that the CaSR-claudin-14 pathway is activated in an induced stone model both *in vitro* and *in vivo*.

### Calcium oxalate-induced CaSR activation increases claudin-14 expression via STAT3

**FIGURE 2:**
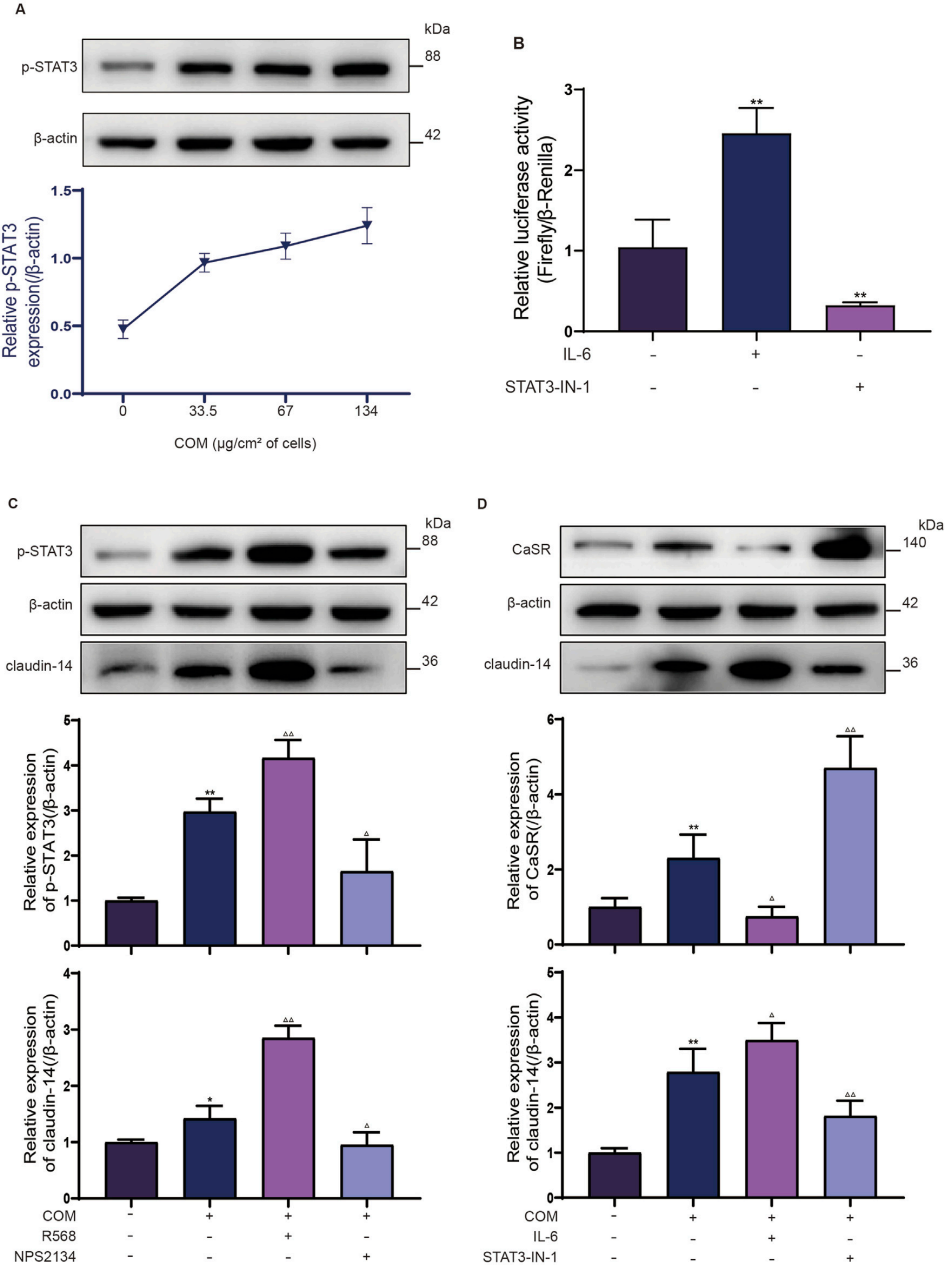
STAT3 is a transcription factor involved in CaSR regulation of claudin-14. **(A)** NRK-52E cells were induced with COM (0, 33.5, 67 or 134 *μ*g/cm2 of cells) for 6 h, after which p-STAT3 protein expression was examined by Western blotting. **(B)** Luciferase activity of NRK-52E cells specifically activated with IL-6 or inhibited with STAT3-IN-1. **(C)** The protein expression of p-STAT3 and claudin-14 in NRK-52E cells specifically activated with R568 or inhibited with NPS2134. **(D)** Protein expression of CaSR and claudin-14 in NRK-52E cells specifically activated with IL-6 or inhibited with STAT3-IN-1. The data are presented as the mean ± standard deviation of three independent experiments. *p < 0.05 vs the control group; **p < 0.01 vs the control group; ^△^ *p* < 0.05 vs the COM group (the second band); ^△△^ *p* < 0.01 vs the COM group. Statistical analyses were performed by one-way analysis of variance. COM, calcium oxalate monohydrate; CaSR, calcium sensing receptor; STAT3, signal transducer and activator of transcription 3.

Subsequently, to validate the role of STAT3 in the CaSR-claudin-14 pathway, p-STAT3 protein expression in NRK-52E cells exposed to varying concentrations of COM (0, 33.5, 67, or 134 *μ*g/cm2 of cells) for 6 h was determined by Western blotting. The response revealed a dose-dependent change in p-STAT3 levels. The protein expression of p-STAT3 in the NRK-52E cells increased by 1.3-fold after the cells were exposed to 134 *μ*g COM crystals/cm2 of cells (Figure 2A).

To further confirm the upregulation of claudin-14 expression by CaSR through STAT3 activation, NRK52E cells were induced with COM and incubated with the CaSR activator R568 (30 μM) or the CaSR inhibitor NPS2143 (10 μM) for 6 h. Subsequently, the phosphorylation status of STAT3 and claudin-14 expression were evaluated. In line with prior observations, CaSR activation by R568 led to a 1.4-fold increase in claudin-14 expression, whereas CaSR inhibition by NPS2143 resulted in a 50% decrease in claudin-14 expression (Figure 2C). Furthermore, a notable increase in STAT3 phosphorylation was noted upon CaSR activation (Figure 2C), while a significant decrease was observed upon CaSR inhibition (Figure 2C). Moreover, In conjunction with COM-induced CaSR activation, NRK52E cells were exposed to IL-6 (100 ng/mL), a specific activator of STAT3, or STAT3-IN-1 (30 μM), an inhibitor, for 6 h to evaluate CaSR and claudin-14 expression. As expected, IL-6 increases claudin-14 expression by 1.2-fold in NRK52E cells and correspondingly inhibits the expression of CaSR, whereas STAT3-IN-1 inhibited 60% of claudin-14 expression and further increased CaSR expression. (Figure 2D). These findings suggest that CaSR promotes the expression of claudin-14 through the STAT3 pathway.

Finally, our objective was to investigate whether STAT3 directly regulates the expression of claudin-14 b y interacting with the cldn-14 promoter sequence via a dual-luciferase assay. A 2000 bp sequence upstream of the rat cldn-14 gene, which includes the promoter region, was cloned and inserted into the luciferase reporter gene vector. Luciferase activity was subsequently evaluated under conditions of both STAT3 activation and inhibition, with normalization to Renilla luciferase activity. STAT3 activation caused a 2.4-fold increase in firefly luciferase activity compared to that in the control group, whereas STAT3 inhibition led to a 30% reduction in firefly luciferase activity (Figure 2B). These findings indicate a direct interaction between STAT3 and the cldn-14 promoter sequence, suggesting its role in the regulation of claudin-14 expression. In conclusion, these results collectively validate the role of STAT3 as a mediator of CaSR in the regulation of claudin-14.

### CaSR activation signals through the PKA pathway to promote STAT3 phosphorylation

**FIGURE 3:**
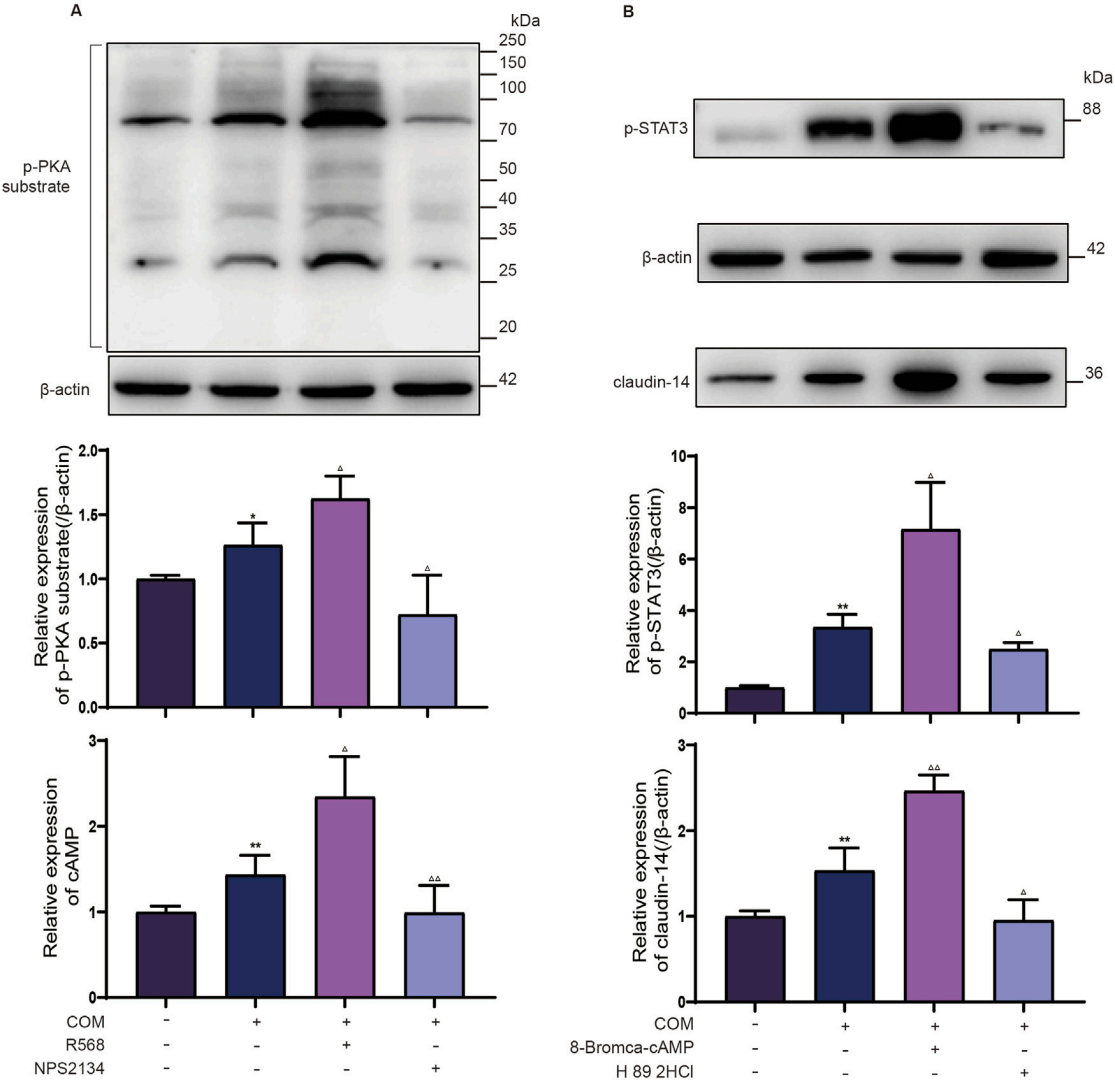
CaSR regulates STAT3 phosphorylation levels through PKA. **(A)** Protein expression of the p-PKA substrate and intracellular cAMP level in NRK-52E cells specifically activated with R568 or inhibited with NPS2134 for CaSR. **(B)** Protein expression of p-STAT3 and claudin-14 in NRK-52E cells specifically activated with 8-Bromca-cAMP or H 89 2HCl inhibited by NPS2134 for PKA. The data are presented as the mean ± standard deviation of three independent experiments. *p < 0.05 vs the control group; **p < 0.01 vs.; ^△^ *p* < 0.05 vs the COM group (the second band); ^△△^ *p* < 0.01 vs the COM group. The control group. Statistical analyses were performed by one-way analysis of variance. COM, calcium oxalate monohydrate; CaSR, calcium-sensing receptor; STAT3, signal transducer and activator of transcription 3; PKA, protein kinase **(A)**.

To determine the involvement of PKA in CaSR-mediated STAT3 phosphorylation, investigators assessed the levels of phosphorylated PKA substrates in distinct NRK-52E cell groups. As depicted in Figure 3A, R568 augmented the phosphorylation levels of proteins ranging from 80 to 100 kDa, whereas NPS2143 negated the increase in phosphorylated proteins in the same range induced by calcium oxalate. This corresponds to the molecular weight of p-STAT3, and in addition, the consistent response of CaSR revealed the possibility that PKA is involved in the induction of STAT3 phosphorylation by CaSR. Similarly, on the basis of the COM-induced increase in intracellular cAMP levels, R568 further increased the intracellular levels of cAMP, whereas NPS2143 counteracted the induction of cAMP by COM. Furthermore, NRK-52E cells were cocultured with the PKA-specific activator 8-Bromca-cAMP (30 μM) and the PKA inhibitor H 89 2HCl (30 μM) in combination with COM for 6 h. Western blot analysis revealed 2.1-fold and 1.6-fold upregulation of the expression of p-STAT3 and claudin-14 proteins in NRK-52E cells upon PKA activation, whereas PKA inhibition halved the protein levels, reducing them to 65% and 50% of their baseline levels, respectively (Figure 3B). This finding suggested that PKA activation facilitates claudin-14 expression through STAT3 activation. In summary, these findings suggest that CaSR-mediated modulation of claudin-14 occurs via the PKA-STAT3 pathway.

### Intervention targeting PKA and STAT3 inhibits the effect of CaSR on kidney stone formation

**FIGURE 4:**
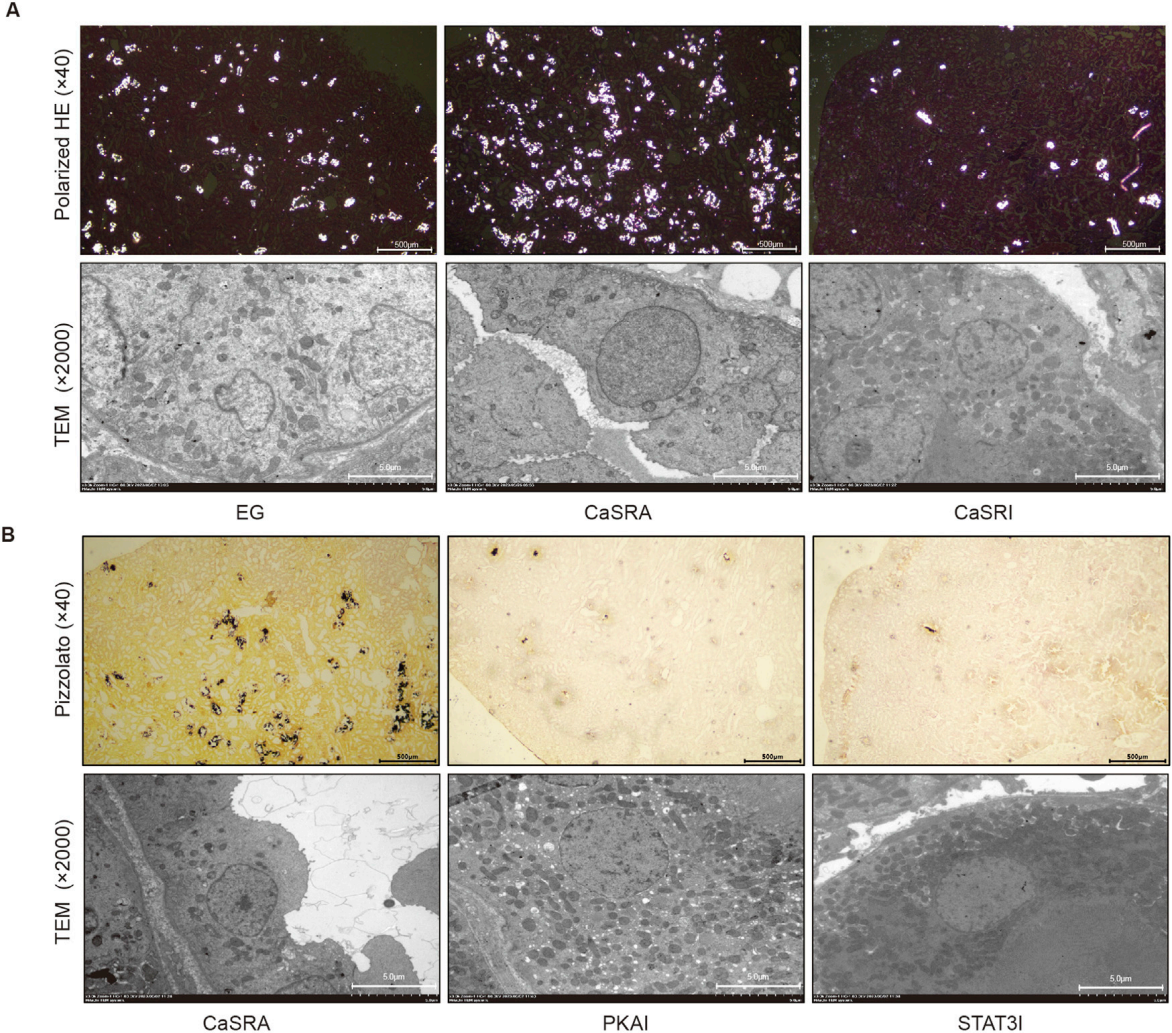
Morphologic distribution of calcium-containing crystals in renal and tubular epithelial cells. **(A)** Representative micrographs of calcium-containing crystalline deposits in renal and tubular epithelial cells of the, E.G., CaSRA and CaSRI groups obtained via hematoxylin-eosin staining, polarized light optical microscopy and transmission electron microscopy. **(B)** Representative micrographs of calcium-containing crystalline deposits in renal and tubular epithelial cells from the CaSRa, PKAI and STAT3I groups obtained via Pizzolato staining and transmission electron microscopy.

**FIGURE 5:**
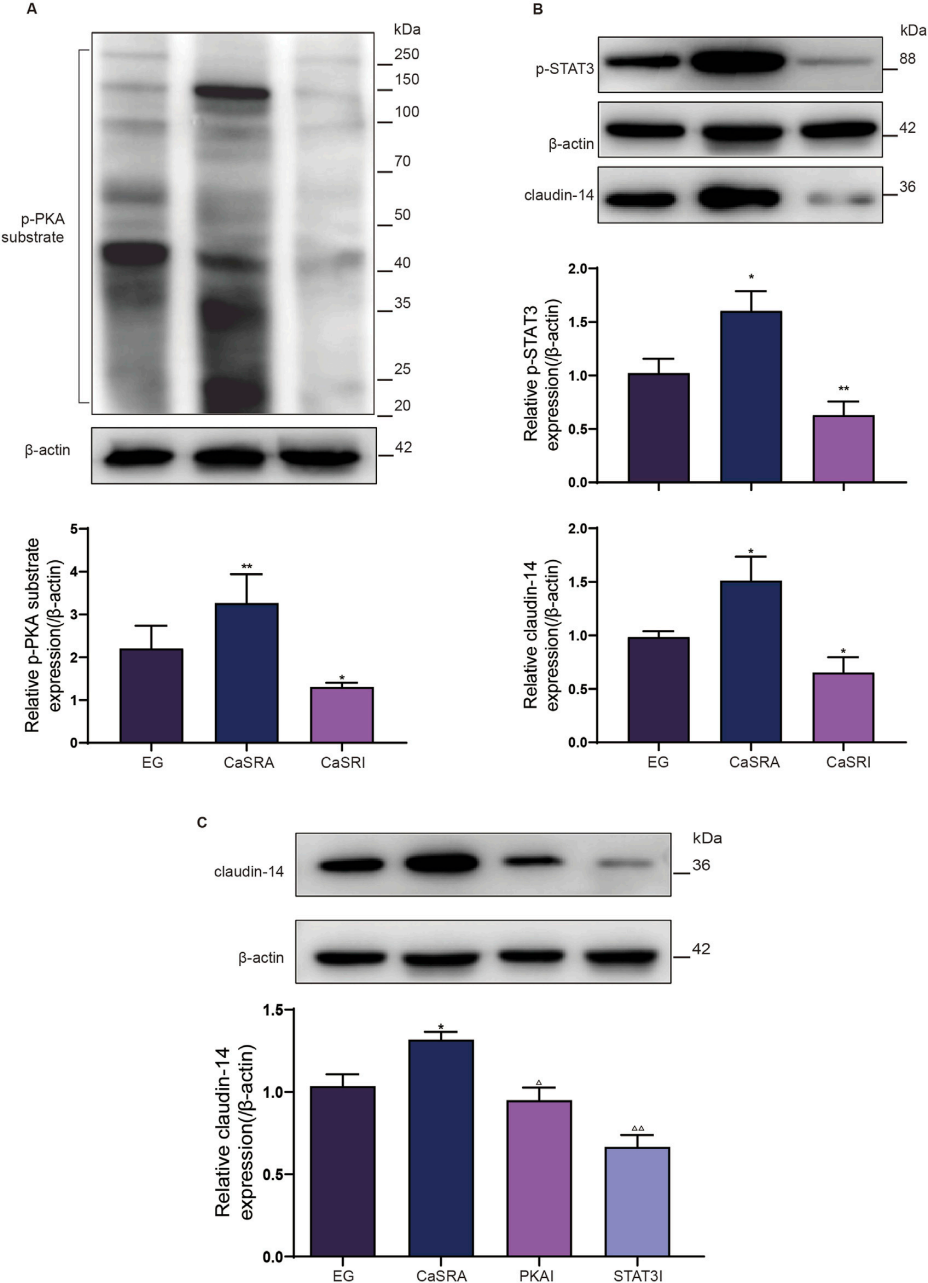
Protein expression of p-PKA substrate, p-STAT3 and claudin-14 in kidney of rats in each group. **(A)** Protein expression of p-PKA substrate in kidney of, E.G., CaSRA and CaSRI groups. **(B)** Protein expression of p-STAT3 and claudin-14 in kidney of, E.G., CaSRA and CaSRI groups. **(C)** Protein expression of claudin-14 in kidney of, E.G., CaSRA,PKAI and STAT3I groups. The data are presented as the mean ± standard deviation of three independent experiments. *p < 0.05 vs E.G., group; **p < 0.01 vs E.G., group; ^△^ *p* < 0.05 vs CaSRA group; ^△△^ *p* < 0.01 vs CaSRA group. Statistical analyses were performed by one-way analysis of variance. COM, calcium oxalate monohydrate; CaSR, calcium sensing receptor; STAT3, signal transducer and activator of transcription 3; PKA, protein kinase **(A)**.

Previous research has shown that ethylene glycol-activated CaSR modulates claudin-14 expression via the PKA-STAT3 pathway *in vitro*. The present study aimed to determine whether these *in vivo* results were recapitulated in NRK-52E cells and to elucidate the involvement of this pathway in stone formation. Renal histopathology indicated that specific activation of CaSR exacerbated crystal formation in the kidneys. Conversely, inhibiting CaSR led to a notable decrease in intratubular crystals, yet a considerable presence of black calcium-containing crystals was noted within renal tubular epithelial cells (Figure 4A). Additionally, consistent with the *in vitro* data, the protein expression of p-PKA, p-STAT3, and claudin-14 was markedly greater in the CaSRA group than in the, E.G., group, whereas the upregulation of these proteins was diminished in the CaSRI group (Figure 5).

To further elucidate the role of PKA and STAT3 in the CaSR-claudin-14 pathway, we treated a rat model of ethylene glycol-induced kidney stones by co-administering the CaSR activator R568 along with the PKA inhibitor or the STAT3 inhibitor. The results showed that both H892HCL and Stattic could counteract the stimulatory effect of R568 on the formation of renal crystals. Additionally, similar to NPS-2134, both inhibitors increased the amount of black crystals within renal tubular epithelial cells (Figure 4B). In addition, upon examination of claudin-14 expression levels, the application of PKA and STAT3 inhibitors mitigated the CaSR-induced increase in claudin-14 levels (Figure 5C). To summarize, targeted inhibition of PKA and STAT3 can attenuate the stimulatory impact of CaSR on claudin-14, thus inhibiting kidney stone formation. This finding offers additional *in vivo* validation of the regulatory role of CaSR on claudin-14 through the PKA and STAT3 signaling pathways, consequently impacting stone formation.

### Knockout of *cldn-14* alone cannot inhibit the induction of renal calculi by ethylene glycol

**FIGURE 6:**
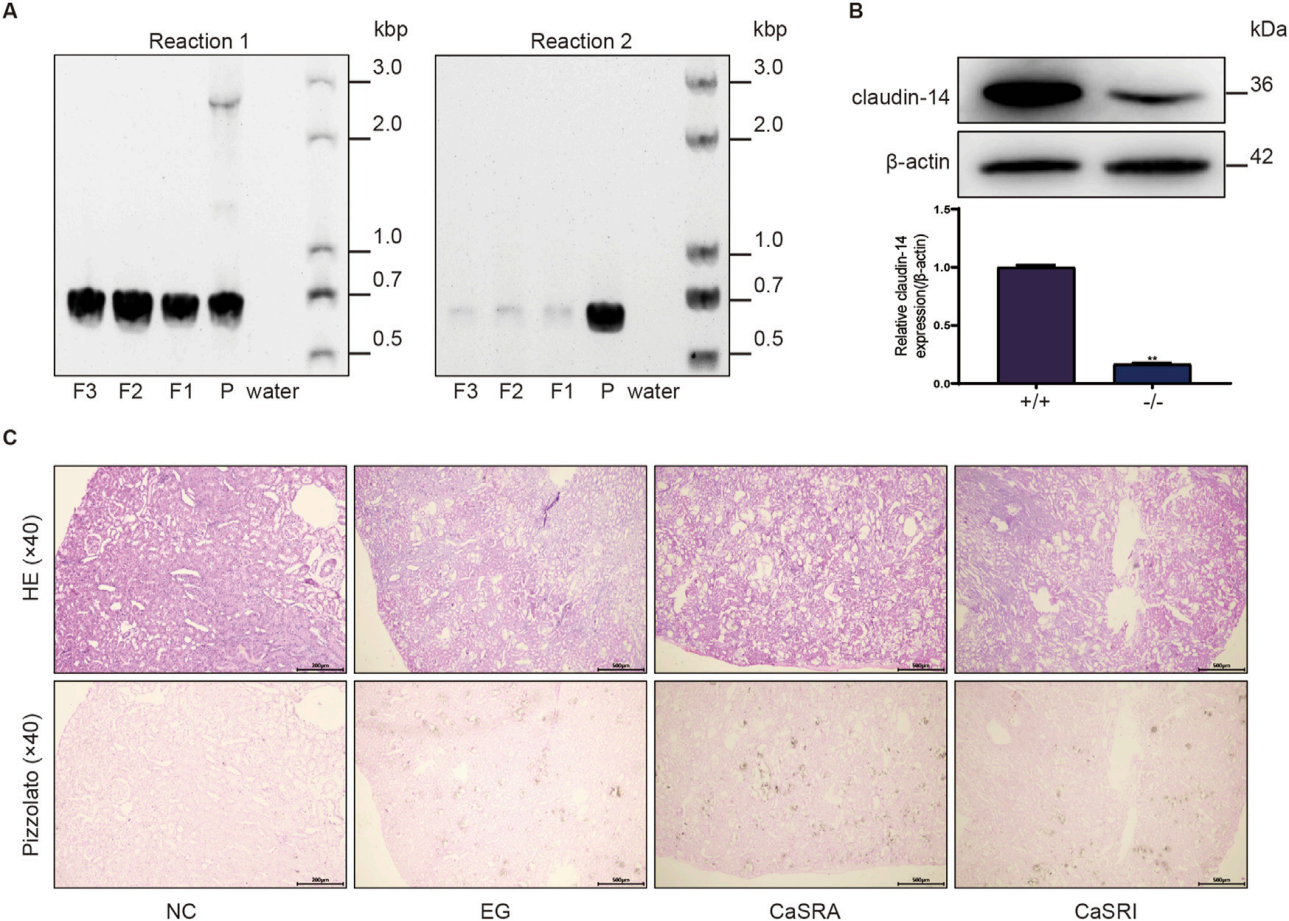
Morphologic distribution of renal calcium oxalate crystals in cldn-14 gene knockout rats. **(A)** Genotyping reactions of parental (P) and offspring (F) rats using specific primers for the coding exon of claudin-14 (Reaction 1) or not containing the claudin-14 coding exon (Reaction 2). **(B)** Protein expression of claudin-14 in the kidneys of wild-type (+/+) or claudin-14 knockout (−/−) rats. The data are presented as the mean ± standard deviation of three independent experiments. **p < 0.05 vs the wild-type group. Statistical analyses were performed by two-tailed unpaired t-tests. **(C)** Representative micrographs of calcium oxalate crystals in the kidneys of the NC, E.G., CaSRA and CaSRI groups, as determined by hematoxylin-eosin and Pizzolato staining.

The above results demonstrate that CaSR activation impacts both claudin14 levels and the formation of kidney stones in rats via the PKA-STAT3 pathway. However, it remains uncertain whether alterations in renal crystal levels stem directly from CaSR activation or result from CaSR modulation of claudin14 via this pathway. To address this question, we constructed cldn-14 gene knockout rats using CRISPR-Cas9 technology and selected 40 male homozygous rats for stone induction and CaSR regulation. Figure 6A shows that the genotyping results for the parental rats revealed three DNA bands (720 bp, 659 bp, and 2,968 bp), suggesting heterozygosity. DNA amplification products from the three selected offspring rats showed only a 720 bp DNA band, confirming the homozygous knockout of the cldn-14 gene. Kidney tissue samples were subsequently obtained from both wild-type and homozygous offspring rats for Western blot (WB) analysis. As depicted in Figure 6B, clear target protein bands were observed in the wild-type rat samples, whereas kidney tissue from homozygous offspring rats showed minimal claudin 14 expression. Unexpectedly, ethylene glycol still induced kidney stone formation in cldn-14 gene knockout rats; however, unlike in wild-type rats, the regulation of CaSR had little effect on stone formation (Figure 6C).

## Discussion

The mechanism of kidney stone formation is complex, and although researchers have elucidated the formation of kidney stones in terms of crystal formation, adhesion, and aggregation, there is currently no clear indication of the initiating factor for stone formation, that is, the markers of the beginning of stone formation (Khan et al., 2016). It is established that the formation of crystals in a solution necessitates that the product of the concentrations of the ions dissolved in the solution reaches a specific value (for example, for COM it is approximately 4 × 10^−9^) (Daudon et al., 2016). In the context of normal human urine, the concentrations of calcium and oxalate ions do not reach the aforementioned value. Nevertheless, the precipitation of calcium oxalate crystals can take place when the ion concentration product exceeds 4 × 10^−9^ in the presence of hypercalciuria or hyperoxaluria. This also elucidates the rationale behind the successful construction of a rat kidney stone model with a diet of ethylene glycol alone. It has been demonstrated that COM-induced oxidative stress promotes the nucleation, aggregation, and retention of crystals and ultimately stone formation by damaging renal tubular epithelial cells (Thamilselvan et al., 2014; Khan, 2006). Furthermore, our study demonstrated that COM-induced CaSR activation promoted stone formation. This suggests that crystal oversaturation may not only be the primary driving force for stone formation, but may also represent a crucial point in the formation process.

In addition to its involvement in diverse pathways, including crystal formation, focusing, and adhesion through oxidative stress and other mechanisms (Thamilselvan et al., 2014; Khan, 2006; Li et al., 2016; Cao et al., 2001), CaSR plays a pivotal role in regulating the body’s water and salt metabolism. Consequently, CaSR exerts a notable influence on urine supersaturation. As previously stated, CaSR plays a multitude of roles within renal tubules. Activation of CaSR in proximal tubules and collecting ducts inhibits urine supersaturation, whereas activation of CaSR at the TAL segment and distal tubules promotes urinary supersaturation, particularly calcium supersaturation, which leads to calcium-containing crystal formation (Blankenship et al., 2001; Gamba and Friedman, 2009; Dimke et al., 2013). As evidenced by our pathological results, the primary sites of stone formation and the primary sites of CaSR and claudin-14 expression are predominantly located in the distal tubules. Therefore, although it is established that COM induces CaSR activation, the imbalance between the “stone-promoting” and “stone-suppressing” effects of CaSR represents a significant area of concern, and further clarification of the “stone-promoting” and “stone-suppressing” effects of CaSR is required. Further clarification is required regarding the specific inducing factors and mechanisms of CaSR’s “stone-promoting” and “stone-suppressing” effects.

This paper describes the “stone-promoting” pathway in which COM promotes stone formation by activating the CaSR-claudin-14 pathway. Furthermore, we emphasize that CaSR influences claudin-14 expression by modulating the phosphorylation status of the transcription factor STAT3 via PKA. CaSR, a G protein-coupled receptor, activates the GTP-binding regulatory protein (G protein) through the secondary messenger cAMP, thereby inducing a response in target cells (Xie et al., 2014; He et al., 2024). In multicellular organisms, the cAMP signal predominantly operates through PKA mediation, facilitating the phosphorylation of downstream proteins (Macías-García et al., 2019; Beebe, 1994). Thus, our study focused on PKA activation by monitoring the phosphorylation of PKA substrates. We validated the ability of CaSR to activate PKA, consistent with prior research findings. Considering the general mechanism of G-protein-coupled receptor activation of PKA (Stefan et al., 2011; Gesmundo et al., 2017), CaSR is hypothesized to potentially activate PKA via the CaSR/Gα/cAMP/PKA pathway. Although this study did not extensively explore this mechanism, it nonetheless illustrates CaSR’s ability to regulate claudin-14 expression through PKA.

In eukaryotic cells, PKA signaling phosphorylates gene regulatory proteins, activating the transcription of target genes (Ha et al., 2010). STAT3 serves as a direct substrate of Janus kinases, playing a pivotal role in transcriptional regulation (Johnson et al., 2018). Upon upstream signaling, tyrosine residues at the C-terminus of STAT3 proteins are phosphorylated, leading to the formation of phosphorylated STAT3 dimers that translocate into the nucleus, thereby modulating the transcription of target genes (Cimica et al., 2011). This study revealed that the phosphorylation of STAT3, akin to that of claudin-14, is regulated by PKA, suggesting that PKA signaling activation of STAT3 participates in claudin-14 expression. Additionally, by cloning a 2000 bp fragment containing the *cldn-14* promoter into a luciferase reporter gene vector and concurrently activating or inhibiting STAT3, we observed corresponding increases or decreases in firefly luciferase activity, indicating that STAT3 may directly bind to the *cldn-14* promoter, thereby participating in claudin-14 regulation. In conclusion, we demonstrated that CaSR-mediated regulation of claudin-14 may be accomplished through the CaSR-PKA-STAT3-claudin-14 pathway. As a G-protein-coupled receptor, CaSR stimulates adenylyl cyclase activity via coupled G-proteins, elevating intracellular cAMP levels and thereby activating cAMP-dependent PKA. PKA signaling phosphorylates STAT3, subsequently leading to the formation of phosphorylated STAT3 dimers that translocate into the nucleus, where they bind to the *cldn-14* promoter, thereby promoting claudin-14 expression.

Our *in vivo* experiments aimed to validate the reproducibility of the *in vitro* findings while assessing the impact of the CaSR-claudin-14 pathway on stone formation. Our results indicate that inhibitors of PKA and STAT3 exhibit efficacy comparable to that of CaSR inhibitors in suppressing ethylene glycol-induced stone formation. This is evidenced not only by a significant reduction in crystal formation but also by a mitigation of renal damage, including diminished renal tubular dilation, reduced epithelial cell swelling, and decreased inflammatory cell infiltration. These findings suggest that targeting the CaSR-claudin-14 pathway may hold promise for the prevention and treatment of kidney stones. Moreover, we generated rats in which the *cldn-14* gene was knocked out via the induction of calcium oxalate stone formation using ethylene glycol, as previously described. Surprisingly, despite cldn-14 gene knockout, ethylene glycol still promoted stone formation in the rats, albeit with a distinction from their wild-type counterparts: selective modulation of CaSR had no significant impact on stone formation. Drawing on the literature and experimental foundations, we propose a plausible explanation: claudin family proteins may interact synergistically or impose constraints on each other, thus achieving greater functional stability. For instance, in the TAL segment, claudin16 and claudin19 form cation channels that facilitate the paracellular transport of cations (Hou et al., 2008). However, this function is also regulated by another member of the claudin family—claudin14. Additionally, studies indicate a direct influence of CaSR activity on the levels of claudin16 (Ikari et al., 2008). Research by Allein et al. also suggested that after knocking out the claudin12 gene, claudin14 expression decreases in a feedback loop to maintain stable calcium ion reabsorption (Plain et al., 2020). Together, these findings suggest the involvement of a potentially intricate signaling pathway involving claudin14 in renal calcium ion metabolism. Whether systemic knockout of claudin14 leads to alterations in the levels of other proteins within the claudin family, thereby affecting calcium ion metabolism, remains undetermined. Nevertheless, this study confirmed at least one finding: a portion of stone formation is mediated through claudin-14; however, mere inhibition of claudin14 may not yield reliable benefits.

Based on the support of the aforementioned findings, we show that COM induces activation of the CaSR during calcium oxalate stone formation in rats and further promotes crystal formation by activating the pro-stone pathway of CaSR: CaSR-PKA-STAT3-claudin-14 to enhance the supersaturation of calcium salts. These studies further augment the understanding of CaSR’s regulation of stone formation, enhance our understanding of the mechanisms of calcium oxalate stone formation, and offer potential targets for therapeutic interventions aimed at stone prevention and management.

## Data Availability

The original contributions presented in the study are included in the article/supplementary material, further inquiries can be directed to the corresponding author.
